# Clinical Accuracy of Non-Contact Forehead Infrared Thermometer Measurement in Children: An Observational Study

**DOI:** 10.3390/children9091389

**Published:** 2022-09-14

**Authors:** Yeon-Mi Kim, Myung-Roul Jang, Ju-Ryoung Moon, Goeun Park, Ye-Jin An, Jeong-Meen Seo

**Affiliations:** 1 Department of Nursing, Heart Vascular Stroke Institute, Samsung Medical Center, Seoul 06351, Korea; 2 Department of Nursing, Cardiac Center, Heart Vascular Stroke Institute, Samsung Medical Center, Seoul 06351, Korea; 3 Biomedical Statistics Center, Research Institute for Future Medicine, Samsung Medical Center, Seoul 06351, Korea; 4 Department of General Surgery, Division of Pediatric Surgery, Samsung Medical Center, Sungkyunkwan University of College of Medicine, Seoul 06351, Korea

**Keywords:** body temperature, thermometers, infrared rays, fever, child

## Abstract

We evaluated the clinical reliability and utility of temperature measurements using no-contact forehead infrared thermometers (NCFITs) by comparing their temperature measurements with those obtained using infrared tympanic thermometers (IRTTs) in children. In this observational, prospective, and cross-sectional study, we enrolled 255 children (aged 1 month to 18 years) from the pediatric surgery ward at a tertiary medical center in Korea. The mean age of the children was 9.05 ± 5.39 years, and 54.9% were boys. The incidence rate of fever, defined as an IRTT reading of ≥38.0 °C, was 15.7%. The ICC coefficient for the assessment of agreement between temperatures recorded by the NCFIT and IRTT was 0.87, and the κ-coefficient was 0.83. The bias and 95% limits of agreement were 0.15 °C (−0.43 to 0.73). For an accurate diagnosis of fever (≥38 °C), the false-negative rate was much lower, but the false-positive rate was higher, especially in 6-year-old children. Therefore, NCFITs can be used to screen children for fever. However, a secondary check is required using another thermometer when the child’s temperature is >38 °C. NCFITs are proposed for screening but not for measuring the temperature. For the latter, an accurate and reliable thermometer shall be used.

## 1. Introduction

Body temperature is an essential indicator of the patient’s general condition [1]. Inaccurate measurements of the body temperature can mislead the diagnosis of febrile disease. Thus, the exact body temperature measurement is necessary for clinical settings [2]. To successfully screen children with elevated temperatures, it is essential that the body temperature is measured accurately and the thermometer outputs are correctly interpreted [3]. However, there is no single ideal way to measure a child’s body temperature [4]. Because the body temperature of children is higher than that of adults and its normal range varies with age [5], the use of different thermometers should be considered according to the child’s age.

The Italian Pediatric Society suggests using a tympanic, contact, non-contact infrared thermometer in children aged ≥ 4 weeks [6]. An infrared tympanic thermometer (IRTT) is often used in children because its use is easy and rapid, and it shows a value close to the rectal temperature [7]. However, its use is restricted to children aged < 2 years because of the anatomy of the external auditory meatus [8], and its accuracy is affected by the presence of earwax in this structure [9]. In addition, using an IRTT can be stressful for children, parents, and nurses because direct contact with the thermometer disturbs sleep or provokes anxiety and fear in the child. Using a non-contact forehead infrared thermometer (NCFIT) can overcome these challenges.

The non-contact infrared thermometer was approved in 2004 as a Food and Drug Administration class II medical device (21 CFR 880.291) [10]. It measures the temperature of various body parts without the need for physical contact. In particular, the forehead is recommended as the target site for such thermometers because the temporal artery, which is used for temperature measurement, receives blood from the carotid artery and reflects the core body temperature [6]. The NCFIT is easy to use, has a low possibility of cross-infection, and does not cause discomfort, such as disturbed sleep [6,11]. Moreover, the use of NCFITs during pandemics, such as the coronavirus disease 2019 pandemic, was found to be effective in decreasing cross-infection.

The ISO 80601-2-56 [5] and ASTM E1965-98 [12] are both Food and Drug Administration-approved voluntary consensus standards used by thermometer manufacturers to evaluate the accuracy and effectiveness of IRTTs and NCFITs in clinical settings by performing a clinical study [3]. Based on these standards and several clinical studies [7,13,14,15], IRTTs were adopted as clinical thermometers. However, NCFITs have not yet been adopted as clinical thermometers because the correlation of NCFITs to the core temperature measured by clinical thermometers has not been confirmed [13,16]. In addition, the findings of studies on the accuracy of NCFITs are not in complete agreement [16,17,18,19,20,21,22,23]. The sensitivity of the NCFIT models for detecting a temperature > 38.0 °C in adults and children is 0–69% [3] and 60%, respectively [18]. These findings suggest that while NCFITs may be acceptable for children without fever, their use can lead to major problems for children with fever. Therefore, the accuracy and credibility of NCFITs should be thoroughly evaluated before using them as a practical measurement device.

Thus, this study aimed to evaluate the accuracy of NCFITs by age compared with that of IRTTs, which are regarded as the gold standard for body temperature measurement in children [24]. It also aimed to identify possible scenarios where NCFITs can be used in clinical settings.

## 2. Materials and Methods

### 2.1. Study Design and Participants

We conducted an observational, prospective study at our institution from 20 December 2019 to 7 June 2020 to assess the clinical accuracy and consistency of NCFITs by age by comparing them with those of IRTTs. The study population comprised children admitted to the pediatric surgery unit with the following inclusion criteria: (1) aged 1 month to 18 years; (2) had no injury in the external auditory meatus and head; (3) had no infection or eruption on the skin of the forehead; (4) had not undergone cold or heat therapy; (5) and were not using barbiturates, thyroid preparations, antipsychotics, or had recent immunizations [25].

### 2.2. Measurements

The following thermometers were used: NCFIT (Hubdic Thermofinder S2, HFS-710, Gyeonggi-do, Korea) and IRTT (Braun ThermoScan 7, IRT 6520, Chihuahua, Mexico). Each thermometer was referred to the Korea Calibration & Technology Institute before data collection. The error range of the NCFIT was ±0.2 °C between 34.0 °C and 42.5 °C, and that of the IRTT was ±0.2 °C between 35.0 °C and 42.0 °C. Thermometers were prepared according to the manufacturer’s instructions. These thermometers were kept in the testing room for 10–30 min before taking the temperature measurements to allow them to adjust to the environment, as described in their user manuals [5,25].

### 2.3. Data Collection/Procedure

All of the data were collected by a research nurse who was well acquainted with the use of each thermometer. The body temperature of the children was measured using both NCFIT and IRTT. The readings were obtained two times within 1 min for each child, and the average value was calculated. However, for babies aged < 90 days, the readings were obtained three times according to the manufacturer’s directions. To homogenize the indoor temperature, humidity, and measurement time, the hospital room conditions were maintained at 25–27 °C and 40–60% humidity, and the body temperatures were measured between 1 pm and 5 pm. The body temperature, sex, and age of children were recorded.

#### 2.3.1. Measurement Using the NCFIT

**Figure 1:**
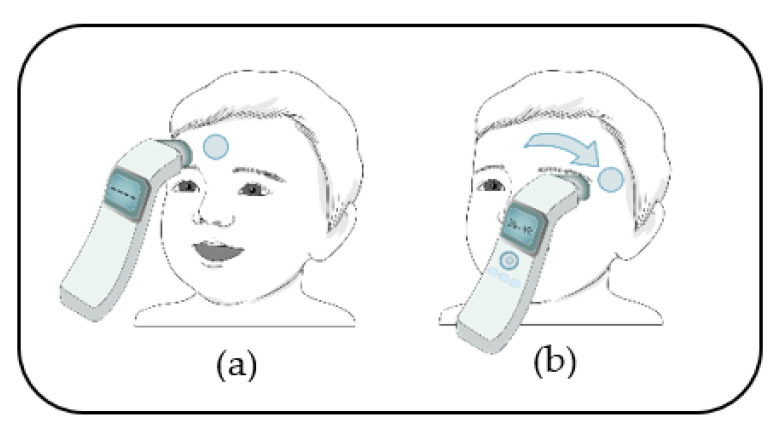
Measurement using the NCFIT. (**a**) The sensing area of the NCFIT was positioned at a distance of 2–3 cm from the center of the forehead at a right angle, and then the measurement button was pushed. (**b**) The scan was performed slowly toward the left temple for 3–4 s with the button being pushed until the measurement completion sound was obtained. The temperature was displayed on the screen once the measurement was complete.

The left forehead was used for temperature measurement. First, the whole forehead was exposed; if there was sweat, it was gently absorbed using gauze. Then, the NCFIT was positioned 2–3 cm above the center of the forehead (Figure 1a). Next, the research nurse pushed the button of the NCFIT and scanned slowly for 3–4 s while moving toward the left temple (Figure 1b). After the measurement, the researcher recorded the values.

#### 2.3.2. Measurement Using the IRTT

To test the accuracy of the two thermometers, the left ear was used for comparison of the output temperature obtained by temperature measurement of the same body part [5]. A disposable probe cover was fitted to the thermometer, and the external auditory meatus was pulled in the posterosuperior direction (posteroinferior for those who were aged < 2 years) to make it straight. Then, the thermometer was inserted completely, and the body temperature was measured.

#### 2.3.3. Readings across Instruments

For both instruments, the unit of measurement was centigrade. All of the figures were recorded to the nearest tenth of a degree.

### 2.4. Statistical Analysis

This study used the SAS 9.4 version (SAS Institute, Cary, NC, USA) and R package version 4.1.3 (http://www.R-project.org, accessed on 7 June 2022); R Project for Statistical Computing, Vienna, Austria) for data analysis. The statistical significance level was set at <0.05. The general characteristics of the participating children and body temperature were recorded, and the frequency, mean, and standard deviation (SD) of the temperatures were calculated. A paired *t*-test was used to calculate the mean difference between the body temperature recordings of the NCFIT and IRTT. To confirm the consistency between NCFIT with the IRTT output temperatures, single-measure intraclass correlation coefficient (ICC) using a two-way mixed model, Kappa coefficient, and Bland–Altman plot were analyzed. The Kappa statistics used a categorical variable of ≥38 °C to evaluate the agreement between the two methods for diagnosing fever. The accuracy of fever detection was described using a 2 × 2 cross table of IRTT and NCFIT. The sensitivity, specificity, positive predictive value (PPV), negative predictive value (NPV), and accuracy were calculated based on the IRTT values at 38 °C. The sensitivity, specificity, predictive values, and accuracy were calculated using the following equations [26]:

$$\mathrm{Sensitivity}=\frac{\#\;of\;subject\;where\;\left(\mathrm{IRTT}\geq 38\;{}^{\circ}C\;and\;\mathrm{NCFIT}\geq 38\;{}^{\circ}C\right)\;}{\;\#\;of\;subject\;where\;\left(\mathrm{IRTT}\geq 38\;{}^{\circ}C\right)}$$

$$\mathrm{Specificity}=\frac{\#\;of\;subject\;where\;(\mathrm{IRTT}<38\;{}^{\circ}C\;and\;\mathrm{NCFIT}<38\;{}^{\circ}C)\;}{\#\;of\;subject\;where\;(\mathrm{IRTT}<38\;{}^{\circ}C)}$$

$$\mathrm{PPV}=\frac{\;\#\;of\;subject\;where\;\left(\mathrm{IRTT}\geq 38{}^{\circ}C\;and\;\mathrm{NCFIT}\geq 38\;{}^{\circ}C\right)\;}{\#\;of\;subject\;where\;\left(\mathrm{NCFIT}\geq 38\;{}^{\circ}C\right)}$$

$$\mathrm{NPV}=\frac{\;\#\;of\;subject\;where\;of\;(\mathrm{IRTT}<38\;{}^{\circ}C\;and\;\mathrm{NCFIT}<38\;{}^{\circ}C)\;}{\#\;of\;subject\;where\;(\mathrm{NCFIT}<38\;{}^{\circ}C)}$$

$$\mathrm{Accuracy}=\frac{\#\;of\;subject\;where\;\left(\mathrm{IRTT}\geq 38\;{}^{\circ}C\;and\;\mathrm{NCFIT}\geq 38\;{}^{\circ}C\right)\;or\;(\mathrm{IRTT}<38\;{}^{\circ}C\;and\;\mathrm{NCFIT}<38\;{}^{\circ}C)\;\;\;}{\;\#\;of\;subject\;where\;\left(\mathrm{NCFIT}\geq 38\;{}^{\circ}C\right)\;or\;(\mathrm{NCFIT}<38\;{}^{\circ}C)}$$

### 2.5. Sample Size

The desired effect size was calculated using the ICC of the two thermometers [27]. Setting the expected value of ICC (ρ1) as 0.80 [28] and the minimum acceptable value of ICC (ρ0) as 0.70 for a “good” result [29]; α = 0.05 (two-tailed); 1 − β = 0.9 [30], a sample size of 198 children was calculated to be sufficient for this study [31]. The target sample size was 248 children, considering a dropout of 20%.

## 3. Results

### 3.1. Participant Characteristics

A total of 255 children were enrolled in this study, and no participants dropped out of the study. Of these 255 participants, 140 (54.9%) were boys, and the average age was 9.69 ± 5.03 years. The gender distribution and the mean age by age groups were calculated (Table 1). Among the participants, 40 (15.7%) children with a temperature ≥ 38.0 °C, whose body temperature was measured using the IRTT, were diagnosed with fever. In addition, 44 (17.3%) children had a body temperature ≥ 38 °C as measured using the NCFIT (Table 1).

### 3.2. Agreement between NCFIT and IRTT

Table 2 compares the NCFIT with the IRTT mean temperatures and mean differences. The mean temperatures measured using the NCFIT and the IRTT were 37.24 ± 0.62 °C and 37.36 ± 0.66 °C, respectively. Temperatures recorded using the NCFIT had a significantly lower mean difference compared to the temperature measured using the IRTT (−0.14 ± 0.29 °C, *p* < 0.001). NCFIT temperatures also had a significantly lower mean difference compared to the IRTT temperatures in all age subgroups (Table 2).

**Figure 2:**
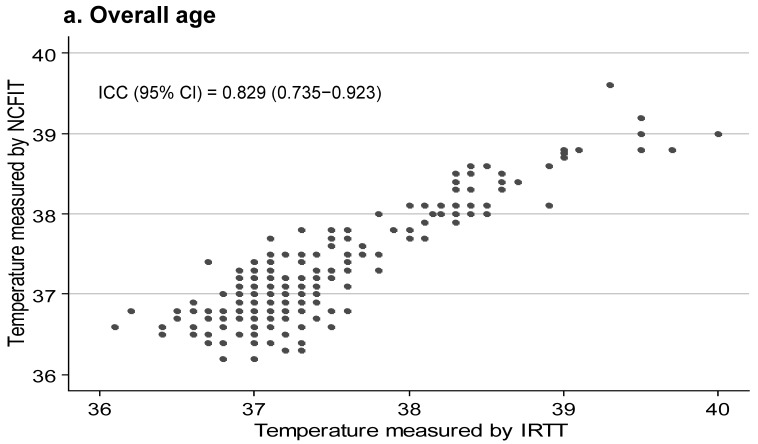

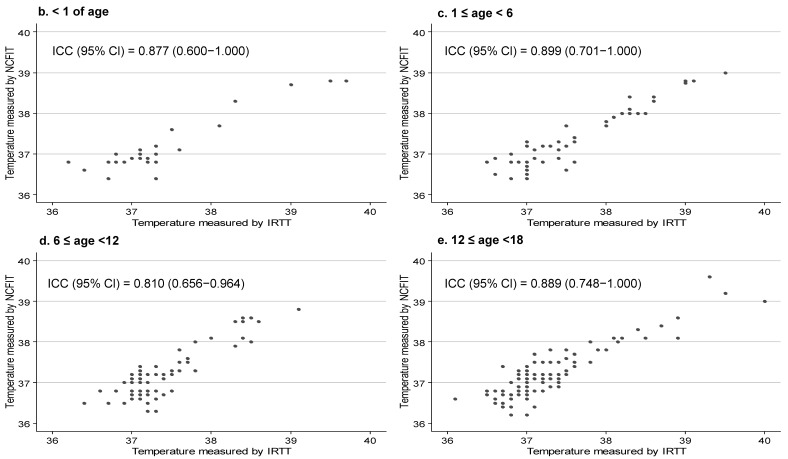
Agreement between NCFIT and IRTT by intra-class correlation coefficient. (**a**) Overall, (**b**) <1 year of age, (**c**) 1–5 years of age, (**d**) 6–11 years of age, and (**e**) 12–18 years of age.

The agreement between the temperature recorded by NCFIT and IRTT by ICC was 0.87 (95% CI; 0.75–0.95). Moreover, in all of the age subgroups, ICC was 0.80 was higher, indicating near complete agreement (Table 2; Figure 2). Furthermore, the Kappa coefficient was greater than 0.80 in all age subgroups except for that the under-1-year age group. These findings indicate near complete agreement. In the under-1-year age group, the Kappa was 0.75 (95% CI; 0.43–1.00), indicating moderate agreement.

**Figure 3:**
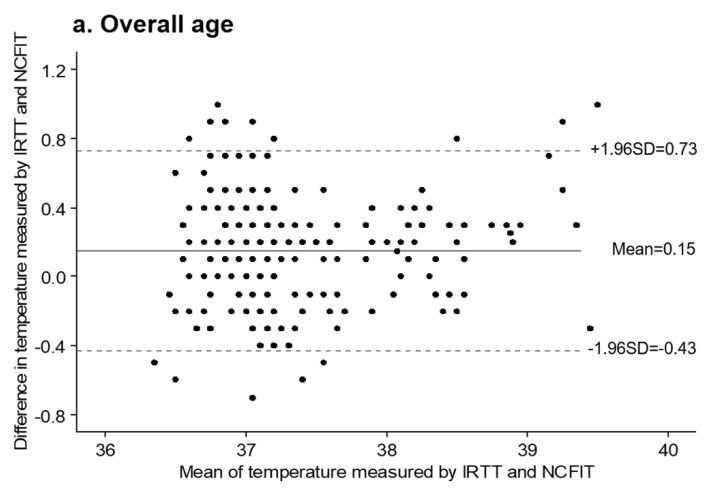

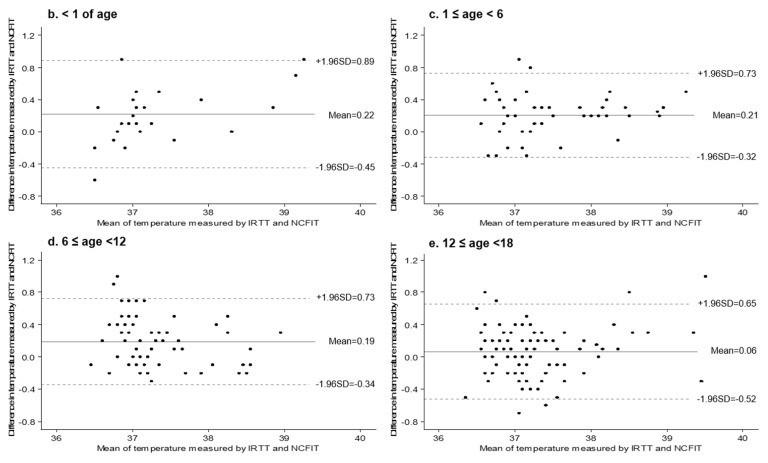
Agreement between NCFIT and IRTT using Bland–Altman plots. (**a**) Overall age, (**b**) <1 year of age, (**c**) 1–5 years of age, (**d**) 6–11 years of age, and (**e**) 12–18 years of age. Bland–Altman plots of difference indicate the mean temperature difference and 95% limits of agreement: comparison of the difference between each NCFIT and IRTT paired temperature measurement. The middle horizontal line represents the mean difference in these two measurements, and the other horizontal lines represent 2SDs above and below the mean difference.

The consistency of the measurements by the two thermometers was determined using the Bland–Altman plot (Figure 3). The body temperature measurements using NCFIT and IRTT were in good agreement. The mean difference (bias) and 95% limits of agreement were 0.15 °C (−0.43 to 0.73) for the overall age group (Figure 3a), 0.22 °C (−0.45 to 0.89) for the <1-year age group (Figure 3b), 0.21 °C (−0.32 to 0.73) for the 1–5 years age group (Figure 3c), 0.19 °C (−0.34 to 0.73) for the 6–11 years age group (Figure 3d), and 0.06 °C (−0.52 to 0.65) for the 12–18 years age group (Figure 3e). Overall, 95.6% of participants in the overall age group, 96.2 % of the <1-year age group, 96.0% of the 1–5 years age group, 97.6% of the 6–11 years age group, and 94.8% of the 12–18years age group were each within the 95% limit of agreements. When comparing the measurements between the two thermometers, the bias for all age groups ranged from 0.06 °C to 0.22 °C, which indicated an acceptable level (±0.3 °C) of bias between the thermometers.

### 3.3. Validity of Fever Detection Based on 38.0 °C of IRTT

The sensitivity, specificity, PPV, and NPV of the NCFIT measurement were 0.81 (95% CI: 0.70–0.93), 0.98 (95% CI: 0.96–0.99), 0.90 (95% CI: 0.80–0.99), and 0.96 (95% CI: 0.93–0.98), respectively, in the overall age group (Table 3). The sensitivity was 0.66 (95% CI: 0.28–1.00) and 0.76 (95% CI: 0.56–0.96) for children aged <6 years, i.e., the <1-year and 1–6 years age groups, respectively, showing a rather low sensitivity. In contrast, for children aged >6 years, the sensitivity was >0.90. The specificity was 0.98 (95% CI: 0.96–0.99) in the overall age group, and the lowest specificity was observed for the 6–11 years age group (0.94 [95% CI: 0.93–1.00]). The PPV (0.90 [95% CI: 0.80–0.99]) and NPV (0.96 [95% CI: 0.93–0.98]) were also high in the overall age group. The PPV was the lowest in the 6–11 years age group, being 0.81 (95% CI: 0.59–1.00). The NPV was the lowest in the 1–5 years age group, being 0.89 (95% CI: 0.19–0.99) (Table 3).

## 4. Discussion

NCFITs are preferred in clinical settings because of their ease of use, but their scientific benefits have yet to be proven consistently. Therefore, this study aimed to evaluate the accuracy of an NCFIT by age, compared with that of an IRTT, for children aged < 18 years.

In the present study, the temperature measured using the NCFIT was 0.14 °C lower than that using the IRTT in the overall age group. This finding is similar to those of previous studies showing that NCFITs record temperatures 0.1 °C lower than those recorded using IRTTs in children aged < 5 years [32] and describing that NCFIT measurements were 0.17 ± 0.48 °C lower than IRTT measurements in participants aged < 18 years [33]. However, a previous study reported that the mean forehead temperature was 2.07 ± 0.31 °C lower than the mean tympanic temperature in adults [34]. In a study by Sullivan et al. that compared an axillary thermometer with six commercialized NCFITs in 1113 adults, the individual differences ranged from −3 °C to 2 °C in only extreme cases, with a majority of the differences ranging from −2 °C and 1 °C [3]. Such large variability in the NCFIT measurements may be due to the study design, subject characteristics, and device characteristics. NCFITs measure body temperature by detecting infrared radiant energy from the skin surface of the forehead. Usually, the measured temperature of the forehead skin surface is lower than the reference body site temperature. Therefore, manufacturers typically use a proprietary algorithm and hardware design features to compensate for the difference between the forehead’s skin surface temperature and the body part’s temperature. The algorithms used to adjust temperature can be affected by proprietary software algorithm errors, user errors, physiological variability, and environmental factors, which in turn affect the accuracy of temperature measurements by the NCFIT [3]. Our research protocol was designed to minimize user errors as one examiner and inaccuracy due to environmental factors. In a real clinical setting, the error in the body temperature measured using NCFITs may increase unless the affecting factor is considered in the proprietary algorithm.

Many studies have analyzed the consistency of thermometers using Pearson’s correlation coefficient. One study that was conducted on participants aged between 6 months and 15 years showed a significant correlation between IRTT and NCFIT temperatures (r = 0.70, *p* < 0.01) [33], and another on participants aged 1–48 months described a high correlation between the rectal and forehead temperatures (r = 0.95, *p* < 0.001). However, the ICC is recommended for the evaluation of consistency rather than a correlation coefficient because it comprises both correlation and bias [29]. In the present study, the ICC between the NCFIT and IRTT in the overall age group was between 0.81 and 0.89, and it showed relatively high consistency. This finding is similar to that of a study reporting an ICC of 0.81 (*p* < 0.01) between NCFIT and IRTT temperature measurements among young adults [28].

The present study analyzed the consistency of both thermometers using Bland–Altman plots and the ICC. The Bland–Altman plot analysis enabled the identification of the systematic difference (error) between the two thermometers. In this analysis, the mean difference was the estimated bias, and 95% limits of agreement were represented as the mean difference ±1.96 SD of the difference to compare the mean distribution between the data sets. If bias is close to 0 and the 95% limits of agreement are narrow, the two devices may be used interchangeably [35]. The IOS 80601-2-56 [5] and ASTM E1965 standards [12] require the bias to be within ±0.3 °C. The bias for all age groups in the present study ranged from 0.06 °C to 0.22 °C, which indicated an acceptable level of bias between the two thermometers. These findings are highly consistent with those of another study that showed the bias between NCFIT and IRTT measurements in children aged < 18 years to range from 0.07 °C to 0.24 °C [33]. However, our results are higher than −0.01 as the bias in children aged ≤ 5 years visiting primary care centers with acute illnesses [8]. In addition, our results showed that the 95% limit of agreement between the NCFIT and IRTT measurements ranged from −0.43 to 0.73 in the overall age group, −0.45 to 0.89 in the <1-year age group, −0.32 to 0.73 in the 1–5 years age group, −0.34 to 0.73 in the 6–11 years age group, and −0.52 to 0.65 in the 12–18 years age group. These findings are similar to those of another study that showed −0.77 to 1.11 as the 95% limit of agreement between NCFIT and IRTT measurements in children aged <18 years [33], −1.47 to 1.28 in children aged ≤ 5 years visiting primary care centers with acute illnesses [8], and −1.26 to 0.13 in young adults [28]. Therefore, NCFIT is a potential substitute for IRTT.

The present study also described the diagnostic accuracy based on ≥38.0 °C of NCFIT. The sensitivity was 0.81 (95% CI: 0.70–0.93) in the overall age group, but 66.7 (95% CI: 0.28–1.00) and 0.76 (95% CI: 0.56–0.96) for children aged < 6 years; i.e., the <1-year and 1–6 years age groups, respectively, which was rather low. This value is lower than the NCFIT sensitivity obtained from 1000 readings of 567 children (0.89 [95% CI: 0.83–0.93]) [36] and the sensitivity in a meta-analysis of 19 studies on NCFITs (0.81 [95% CI: 0.66–0.90]) [18]. The specificity was 0.98 (95% CI: 96.3–99.9) in the overall age group, and the lowest specificity was observed for the 6–11 years age group (0.94 [95% CI: 0.93–1.00]). This value is higher than the NCFIT specificity obtained in the study on 567 children (0.75 [95% CI: 0.74–0.76]) [36] and the specificity in the meta-analysis of 19 studies on NCFITs (0.92 [95% CI: 0.80–0.96]) [18]. The PPV (0.90 [95% CI: 0.80–0.99]) and NPV (0.96 [95% CI: 0.93–0.98]) were also high in the overall age group. The PPV was the lowest in the 6–11 years age group, being 0.81 (95% CI: 0.59–1.00). The NPV was the lowest in the 1–5 years age group, being 0.89 (95% CI: 0.19–0.99). These values are higher than the PPV (0.33 [95% CI: 0.31–0.50]) and lower than the NPV (0.98 [95% CI: 0.96–0.98]) reported by Ng et al. [36]. Thus, NCFIT could be a useful instrument to detect fever in children, particularly those who are afraid of body temperature measurement. However, the most valuable factors for the identification of fever are sensitivity and PPV rather than specificity and NPV [17,18,33]. Similarly, in our study, the thermometer with lower sensitivity and PPV, rather than high specificity and NPV, detected fever. Therefore, if the body temperature measured by NCFIT is >38 °C, other methods should be considered to obtain an accurate body temperature [18]. For verifying the temperature of a child suspected of having a fever, an IRTT (in compliance with the standard operating procedure) [37] or the axillary thermometer [5] can be used.

The present study has some limitations. First, the data analyzed were from children who were hospitalized in a tertiary care center. Thus, the findings cannot be generalized. Second, because of their advantages, recent studies have evaluated the accuracy of non-contact thermometers, compared with that of IRTT, in using NCFITs in clinical settings. The final goal of body temperature measurement is to determine the exact core body temperature. Because the tympanum is supplied by the same artery as the hypothalamus, where a thermoregulatory center exists, the tympanic temperature is regarded as the best way to determine the core body temperature [18,37]. However, temperature measurements taken using IRTT could be inaccurate compared with the rectal temperature in some conditions, such as acute and chronic inflammation of the external auditory meatus [18]. Therefore, further studies that refer to reliable core temperatures, such as the pulmonary artery or rectal temperature, are necessary. Third, this study presented the power/sample size calculation for the overall age group but did not present an appropriately powered calculation for the subgroup analysis. Therefore, further studies with appropriate power/sample sizes for subgroups are needed. Fourth, the discrepancy between the measurements of the two thermometers can be explained by the performance, reliability, and uncertainty of the thermometers. The temperature of the black body cavity is typically utilized as the standard temperature for calibration since the emissivity of the canal is extremely close to unity. Accuracy is measured by how closely the thermometer’s measurement result matches the true value of the measurement [38]. Both systematic and random errors can be categorized as measurement errors. Systematic error is the difference between the true value and the mean value of multiple measurements. Random errors are a sign of measurement variability in each individual measurement. However, uncertainty, as well as the disparity between a single observed value and the mean values of numerous measurements, can lead to measurement variability. The calibration equation’s anticipated values, reference standard values, and resolution are among the sources of uncertainty [38]. To act as the blackbody’s standard temperature, a temperature calibrator’s cell was changed. Its calibration equation reduced the systematic errors. The way to reduce random errors is to choose better sensing components or use large samples [39]. In this study, data were collected by one research nurse. There is an advantage in that the data collection consistency can be secured by one collector, but there is a disadvantage in that it cannot be double-checked on the measurements [33]. To compensate for this shortcoming, we analyzed the average value of the two measurements. It is possible that systematic errors have occurred during this process, and we did not consider them. Therefore, further studies applying the calibration equation to improve systematic error are needed. In addition, it may be necessary to conduct repeated studies using one measured value with a sufficiently increased sample size in consideration of bias error. Last, no consensus exists regarding the cutoff point for fever via forehead temperature measurements obtained using NCFITs. The Hong Kong government’s threshold level for fever is 38 °C for IRTTs and 36.0 °C for NCFITs [40]. Lai et al. and Chen et al. suggested temperatures of 36.7 °C [41] and 36.0 °C [38], respectively, to screen for fever. Therefore, a well-designed cutoff point validation study is proposed, along with further studies on the influencing factors and limitations of the offset algorithm in the temperature measurement using NCFITs.

## 5. Conclusions

The consistency between the body temperatures measured using the NCFIT and IRTT was relatively high in children aged < 18 years who were hospitalized in the pediatric surgery unit. For an accurate diagnosis of fever (≥38 °C), the false-negative rate was much lower, but the false-positive rate was higher, especially in 6-year-old children. Therefore, NCFITs can be used to screen children for fever. However, when the body temperature is >38 °C, a secondary check is required using other types of thermometers.

## Figures and Tables

**Table 1:** Participants characteristics (*n* = 255).

	Overall (*n* = 255)	Age < 1 (*n* = 26)	1 ≤ Age < 6 (*n* = 50)	6 ≤ Age < 12 (*n* = 82)	12 ≤ Age < 18 (*n* = 97)
Age (years), (Mean ± SD)	9.05 ± 5.39	0.48 ± 0.27	3.06 ± 1.49	8.84 ± 1.81	14.61 ± 1.72
Sex (boy), *n* (%)	140(54.9)	14 (53.8)	28 (56.0)	42 (51.2)	56 (57.7)
Fever (≥38 °C) detection					
IRTT, *n* (%)	40 (15.7)	4 (15.3)	13 (26.0)	11 (13.4)	12 (12.4)
NCFIT, *n* (%)	44 (17.3)	6 (23.1)	9 (34.0)	10 (12.2)	11 (11.3)

Values are presented as mean ± standard deviation. Abbreviations: IRTT, infrared tympanic thermometer; NCFIT, non-contact forehead infrared thermometer.

**Table 2:** Comparison of tympanic temperatures and non-contact infrared temperatures by age group.

Categories	NCFIT (a)	IRTT (b)	Difference (a–b)	*t(p)*	ICC (95%CI)	Kappa (95%CI)
Overall (*n* = 224)	37.24 ± 0.62	37.39 ± 0.66	−0.14 ± 0.29	−7.99(<0.001)	0.87 (0.75–0.95)	0.82 (0.73–092)
age < 1 (*n* = 26)	37.22 ± 0.70	37.44 ± 0.87	−0.21 ± 0.34	−3.28(0.003)	0.87 (0.60–1.00)	0.75 (0.438–1.00)
1 ≤ age < 6 (*n* = 50)	37.36 ± 0.71	37.57 ± 0.75	−0.20 ± 0.26	−5.41(<0.001)	0.89 (0.70–1.00)	0.81 (0.63–0.98)
6 ≤ age < 12 (*n* = 82)	37.17 ± 0.55	37.36 ± 0.49	−0.19 ± 0.27	−6.35(<0.001)	0.81 (0.65–0.96)	0.83 (0.65~1.00)
12 ≤ age < 18 (*n* = 97)	37.24 ± 0.62	37.30 ± 0.66	−0.06 ± 0.30	−2.07(0.042)	0.88 (0.74–1.00)	0.85 (0.68~1.00)

Values are presented as mean ± standard deviation. Abbreviation: ICC, intra-class correlation coefficient; IRTT, infrared tympanic temperature; NCFIT, non-contact forehead infrared temperature.

**Table 3:** Sensitivity, specificity, PPV, NPV, and accuracy about over 38 °C between non-contact infrared and tympanic. temperatures in different age groups ^†^.

		IRTT		Sensitivity (95% CI)	Specificity (95% CI)	PPV (95% CI)	NPV (95% CI)	Accuracy (95% CI)
		≥38 °C	<38 °C					
**Overall, (*n* = 255)**								
NCFIT	≥38 °C	36	4	$\frac{36}{44}=$ 0.81 (0.70–0.93)	$\frac{207}{211}=$ 0.98 (0.96–0.99)	$\frac{36}{40}=$ 0.90 (0.80–0.99)	$\frac{207}{215}=$ 0.96 (0.93–0.98)	$\frac{243}{255}=$ 0.95 (0.92–0.97)
	<38 °C	8	207					
**age < 1, (*n* = 26)**								
NCFIT	≥38 °C	4	0	$\frac{4}{6}=$ 0.66 (0.28–1.00)	$\frac{20}{20}=$ 1.00 (1.00–1.00)	$\frac{4}{4}=$ 1.00 (1.00–1.00)	$\frac{20}{22}=$ 0.90 (0.78–1.00)	$\frac{24}{26}=$ 0.92 (0.82–1.00)
	<38 °C	2	20					
**1 ≤ age < 6, (*n* = 50)**								
NCFIT	≥38 °C	13	0	$\frac{13}{17}=$ 0.76 (0.56–0.96)	$\frac{33}{33}=$ 1.00 (1.00–1.00)	$\frac{13}{13}=$ 1.00 (1.00–1.00)	$\frac{33}{37}=$ 0.89 (0.79–0.99)	$\frac{46}{50}=$ 0.92 (0.84–0.99)
	<38 °C	4	33					
**6 ≤ age < 12, (*n* = 82)**								
NCFIT	≥38 °C	9	2	$\frac{9}{10}=$ 0.90 (0.71–1.00)	$\frac{70}{72}=$ 0.97 (0.93–1.00)	$\frac{9}{11}=$ 0.81 (0.59–1.00)	$\frac{70}{71}=$ 0.98 (0.95–1.00)	$\frac{79}{82}=$ 0.96 (0.92–1.00)
	<38 °C	1	70					
**12 ≤ age < 18, (*n* = 97)**								
NCFIT	≥38 °C	10	2	$\frac{10}{11}=$ 0.90 (0.73–1.00)	$\frac{84}{86}=$ 0.97 (0.94–1.00)	$\frac{10}{12}=$ 0.83 (0.62–1.00)	$\frac{84}{85}=$ 0.98 (0.96–1.00)	$\frac{94}{97}=$ 0.96 (0.93–1.00)
	<38 °C	1	84					

Abbreviation: IRTT- infrared tympanic temperature; NCFIT- non-contact forehead infrared temperature; PPV-positive predictive value; NPV-negative predictive value. ^†^ Sensitivity, specificity, positive predictive value, negative predictive value, and accuracy were calculated through each equation in the table.

## Data Availability

The datasets created for and/or used in the analysis of the current work are not generally accessible, but are available from the corresponding author on reasonable request.
